# Role of hesperidin/hesperetin against chemotherapy-induced cardiotoxicity: a systematic review of non-clinical studies

**DOI:** 10.1186/s12935-025-03828-5

**Published:** 2025-05-22

**Authors:** Sina Shaernejad, Ali Nosrat, Maryam Baeeri, Nasser Hashemi Goradel, Mirsalim SeyedSadeghi, Mostafa Akbariani, AmirAhmad Arabzadeh, Mahban Rahimifard, Hamed Haghi-Aminjan

**Affiliations:** 1 Students Research Committee, School of Medicine, Ardabil University of Medical Sciences, Ardabil, Iran; 2 Toxicology and Diseases Specialty Group, Pharmaceutical Sciences Research Center (PSRC), Tehran University of Medical Sciences (TUMS), Tehran, Iran; 3 Department of Medical Biotechnology, Maragheh University of Medical Sciences, Maragheh, Iran; 4 Department of Surgery, School of Medicine, Ardabil University of Medical Sciences, Ardabil, Iran; 5 Department of Pharmacology and Toxicology, Faculty of Pharmacy, Tehran University of Medical Sciences, Tehran, Iran

**Keywords:** Doxorubicin, Paclitaxel, Cisplatin, Cyclophosphamide, Epirubicin

## Abstract

Despite the undeniable role of chemotherapeutics in cancer treatment, their administration may be associated with various side effects. Cardiac injury is among the most crucial side effects related to the induction of chemotherapeutic agents. Since the heart is a vital organ, cardiotoxicity often prevents clinicians from continuing chemotherapy. Hesperidin and hesperetin, flavonoids derived from citrus fruits, possess several pharmaceutical properties. This study firstly explores the cardioprotective effects of hesperidin and hesperetin against chemotherapy-induced cardiotoxicity mechanisms, emphasizing their potential as adjunctive therapies. Key literature gaps are identified, and further mechanistic studies will be proposed. The findings underscore the translational potential of these flavonoids, advocating for rigorous preclinical optimization and clinical trials to validate their efficacy and safety. This review lays a foundation for integrating natural compounds into cardioprotective strategies in oncology. A systematic search was conducted in databases (PubMed, Scopus, ISI) until May 2025, according to PRISMA principles. The search terms were chosen according to our research objective and queried in the title and abstract. Following the screening of 82 papers, twelve articles were selected based on our inclusion and exclusion criteria. Based on the evaluated results, chemotherapy adversely affects cardiac tissue, leading to elevated risks of morbidity and mortality. Co-administration of hesperidin and hesperetin with chemotherapy prevents heart injury and preserves cardiac function, maintaining it almost like a normal heart. The protective role of hesperidin and hesperetin is based on their ability to fight free radicals, reduce inflammation, and stop cell death. Nonclinical investigations indicate that hesperidin and hesperetin ameliorate chemotherapy-induced cardiotoxicity. Nonetheless, they may influence the efficacy of anticancer medications, which primarily function by elevating oxidants, inflammation, and apoptosis. This indicates that meticulously designed trials are necessary to evaluate the efficacy and safety of this combination along with the synergistic potential of them in preventing chemotherapy-induced cardiotoxicity while maintaining anticancer effectiveness.

## Introduction

According to previous studies, after cardiovascular disorders, cancer holds the second position as the leading cause of early death globally. Recent studies reveal a significant increase in cancer cases, potentially positioning it as the leading cause of early mortality worldwide [1, 2]. The World Health Organization (WHO) characterizes cancer as a diverse collection of diseases that may arise in almost any organ or tissue when abnormal cells proliferate uncontrollably, extend beyond their usual boundaries to invade neighboring regions, and/or spread to other organs [3]. Cancer remains one of the leading causes of morbidity and mortality worldwide. According to the latest data from GLOBOCAN 2022, an initiative by the International Agency for Research on Cancer (IARC), there were close to 20 million new cancer cases and 10.0 million cancer-related deaths globally in 2022 [4]. Based on a previous study, chemotherapy-related cardiac dysfunction in cancer patients was 63.21 per 1000 person‐years [5]. There are many ways to treat cancer, from the more common ones like surgery, chemotherapy, and radiation therapy to more cutting-edge ones like targeted therapy, ablation therapy, stem cell therapy, natural antioxidants, nanomedicine, sonodynamic therapy, chemodynamic therapy, radionics, and therapies based on ferroptosis [6]. Despite the advancement of novel cancer treatment methodologies, conventional approaches remain the predominant modalities employed globally [7]. Chemotherapy is a commonly utilized method in cancer treatment, noted for its effectiveness in suppressing tumor cells by targeting those with excessively high rates of division and proliferation [8]. However, it can also cause significant adverse effects such as nausea and vomiting, alopecia, cardiotoxicity, chemotherapy-induced peripheral neuropathy, infertility, diarrhea, harm to healthy cells, and organ damage [9]. The heart functions as the engine of the human body; hence, cardiac toxicity represents a significant adverse effect, with any impairment in cardiac function potentially resulting in diminished quality of life or, in extreme cases, mortality. Cardiotoxic consequences that may occur after chemotherapy include hypertension, dilated cardiomyopathy, arrhythmias, myocardial infarctions, and sudden death [10].

Unfortunately, the exact way that chemotherapy hurts the heart is still not fully understood. However, research shows that the formation of free radicals results in oxidative stress, which kills heart cells [11, 12]. Since there isn’t a strong rule for dealing with cardiotoxicity caused by chemotherapy, finding drugs that protect the heart may be crucial for minimizing damage to it during cancer treatment [13]. Hesperidin is a bioflavonoid abundantly present in various citrus fruits, including sweet oranges, mandarins, limes, grapefruits, and lemons [14–16]. Hesperetin is the aglycone derivative of hesperidin [17]. Both hesperidin and hesperetin have diverse pharmacological qualities, including antioxidant, anticancer, and anti-inflammatory effects [18–20]. Numerous studies have demonstrated the advantageous effects of hesperidin and hesperetin consumption in diseases such as diabetes [21, 22], rheumatoid arthritis [23, 24], cancers [25], and several neurological conditions, such as Alzheimer’s disease and Parkinson’s disease [26].

Finally, hesperidin and hesperetin are relevant to the manuscript’s focus due to their well-established bioactive properties, mechanistic alignment with the pathophysiology of chemotherapy-induced cardiotoxicity, and the availability of non-clinical evidence supporting their cardioprotective effects. A systematic review of these compounds can provide valuable insights and pave the way for future clinical studies. This in-depth review looks at whether hesperidin and hesperetin can protect the heart from the damage caused by chemotherapy, since they each play a different role in heart damage. This study conducted a thorough literature review on the functions of hesperidin and hesperetin in chemotherapy-induced cardiotoxicity. It was also explained how chemotherapeutic medications hurt the heart and what roles hesperidin and hesperetin play in this.

## Methods

### Search strategy and information sources

This systematic review was performed in accordance with the Preferred Reporting Items for Systematic Reviews and Meta-Analyses (PRISMA) guideline [27]. A comprehensive literature search was conducted to obtain all relevant studies on “the role of hesperidin/hesperetin on chemotherapy-induced cardiotoxicity” in both medical subject heading (MeSH) or advanced in the electronic databases PubMed/Medline, Scopus, and Web of Science (ISI) using the keywords including; (Hesperidin OR Hesperetin) AND (Heart OR Myocardium OR Myocardial” OR “Cardiac Toxicity” OR “Cardiac Toxicities” OR Cardiomyopathy OR Myocyte OR Cardiopathic OR Cardiopathy OR Cardiotoxicity OR Cardiotoxicities OR Cardiomyocyte OR Cardiac) AND (Bevacizumab OR Avastin OR Mvasi OR Daunorubicin OR Adriamycin OR Doxorubicin OR Idarubicin OR Cisplatin OR Carboplatin OR Paraplatin OR Bleomycin OR Carmustine OR Cyclophosphamide OR Cytophosphane OR Melphalan OR Etoposide OR Etopophos OR Mitomycin OR Vinblastine OR Vinorelbine OR Navelbine OR Paclitaxel OR Taxol OR Docetaxel OR Taxotere OR Procarbazine OR Asparlas OR Epirubicin OR Amethopterin OR Mustine OR Embikhin OR Mechlorethamine OR Oxaliplatin OR Eloxatin OR Cytarabine OR Cytosine OR Fluorouracil OR Adrucil OR Capecitabine OR Xeloda OR Vincristine OR Sunitinib OR Irinotecan OR Chemotherapy) up to May 2025.

### Criteria for eligibility and selection of studies

After acquiring and organizing the articles in the reference management software, duplicates were eliminated. Subsequently, two independent evaluators (Sina Shaernejad and Ali Nosrat) conducted a two-phase screening procedure. The initial screening process evaluated the articles by analyzing their titles and abstracts according to their relevance to the objectives of the current study. In the second screening process, we assessed the full-text articles in the concluding phase according to our inclusion and exclusion criteria. For our research, our inclusion criteria included (1) the articles that focused on the role of hesperidin/hesperetin in chemotherapy-induced cardiotoxicity; (2) peer-reviewed articles; (3) articles that had enough data; and (4) articles that didn’t have any restrictions on the publication year. The study’s exclusion criteria included (a) cardiac hemodynamic data, (b) book chapters, (c) case reports, (d) irrelevant research, (e) letters to the editor, (f) editorials, (g) review articles, (h) posters, and (i) conference papers and abstracts.

### Data extraction process

Two researchers (Sina Shaernejad and Ali Nosrat) assessed each qualifying study and then gathered the following information: (1) Author’s name and publication year; (2) types of models utilized (in-vivo and/or in-vitro); (3) dosage of chemotherapy medications, administration protocol, and method of delivery; (4) effects of chemotherapy agents on cardiac cells and tissues; (5) dosage of hesperidin/hesperetin, administration protocol, and method of administration; and (6) outcomes of concurrent hesperidin/hesperetin and chemotherapy administration.

## Results

### Literature search and screening

**Fig. 1:**
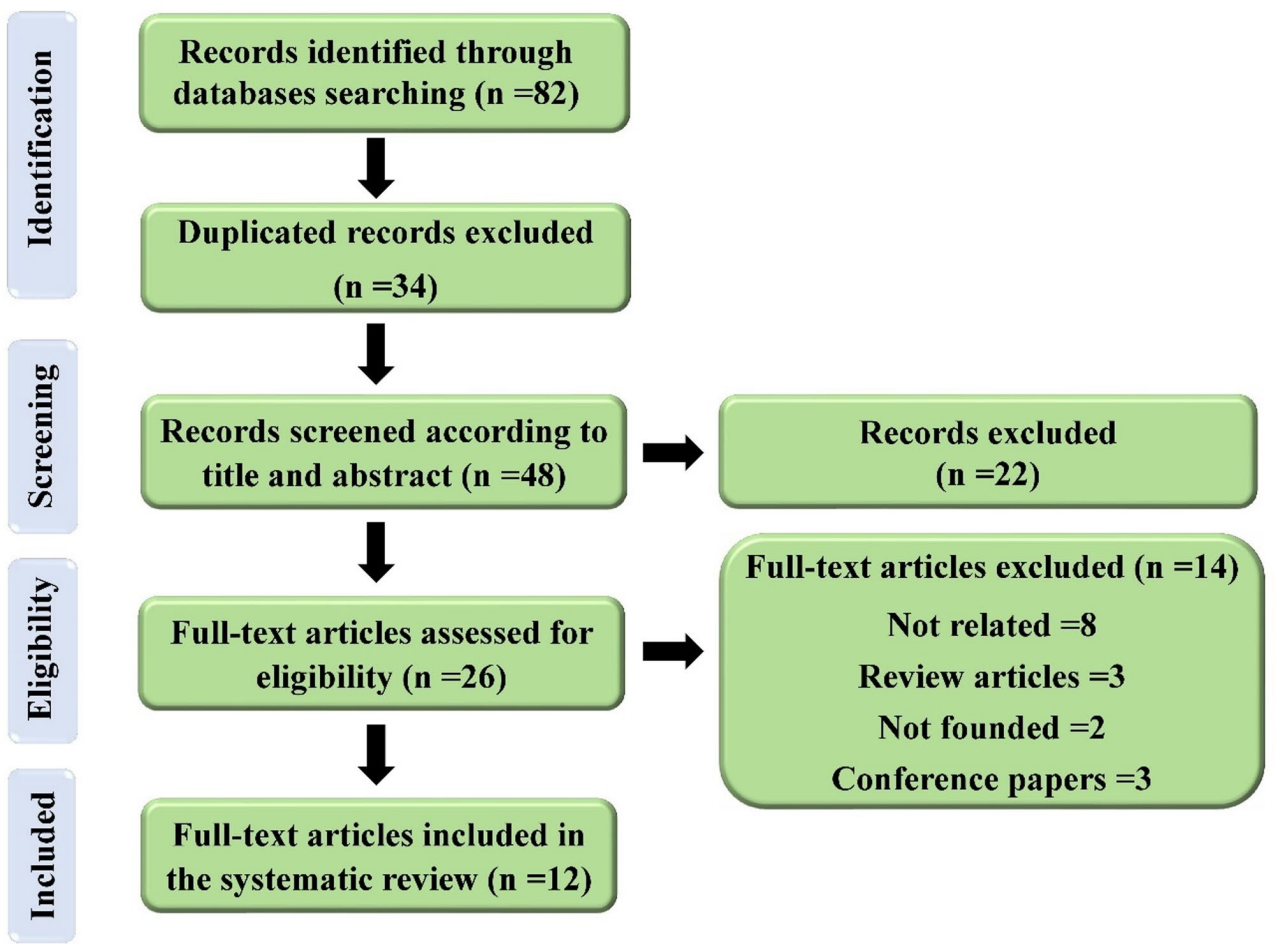
Flowchart illustrating the selection procedure employed in the current investigation

Figure 1 illustrates the procedure for study selection. The elimination process was carried out according to the technique specified in the pervious study [28]. A total of 82 articles were obtained by a systematic search of the specified electronic databases until May 2025. Following the removal of duplicate articles (*n* = 34), the remaining articles (*n* = 48) were assessed according to their titles and abstracts (first screening), resulting in the exclusion of 22 articles. We considered 26 articles suitable for full evaluation, of which 14 articles were removed (8 articles = unrelated, 3 articles = review articles, 2 articles = not founded and 1 article = conference papers). Twelve articles were finally selected for inclusion in this study, adhering to the established criteria for inclusion and exclusion.

### Data extraction

Tables 1 and 2 present additional data from each article, utilizing the methodologies of Sina Shaernejad and Ali Nosrat. Two researchers independently reviewed and agreed on all potential inconsistencies.

**Table 1:** The characteristics hesperidin of included studies

Author, year	Models	Chemotherapy medications (dosage) & Treatment protocol & Route of administration type	Outcomes of chemotherapy medications on cardiac tissue	Hesperidin dosage & protocol of usage & route of administration	Hesperidin co-administration outcomes
Alharbi, F.K., et al.,2023 [29]	In-vivo/ Rat	Doxorubicin (4 mg/kg b.w) & once every week for five consecutive weeks & *ip*	↑Levels of cTn-I, CK-Total, CK-MB, LDH, AST, IFN-γ, IL-1β, & TNF-α, ↓Activity of GPx, catalase & SOD, severe zenker’s degenerations, ↑intercellular spaces with interstitial edema, disarray of the cardiomyocytes, abnormal thickened cardiac muscle, severe vacuolizations, severe thickening of the blood vessels wall, ↑collagen fibers formations, excessive extravasated RBCs, ↑infiltration of inflammatory cells	100 mg/kg b.w/day & 5 days in a week up to five consecutive weeks & *po*	↓ Levels of Troponin I, CK Total, CK-MB, LDH, AST, IFN-γ, IL-1β, & TNF-α, ↑Activity of GPx, catalase & SOD, normal looking cardiomyocytes, slight blood vessel dilatation, individual extravasated RBCs, mild zenker’s degenerations, ↓infiltration of inflammatory cells
Ali, Y. A., et al.,2023 [30]	In-vivo/ Rat	Paclitaxel (2 mg/kg b.w) & twice a week for 6 weeks & *ip*	↑Levels of CK-MB & LDH ↓Activity of GPx & SOD ↑LPO, ↓GSH content, ↑severe hyalinosis & severe leucocytic infiltration	10 mg/kg b.w & twice Weekly & *po*	↓Levels of CK-MB & LDH, ↑Activity of GPx & SOD, ↓ LPO, moderate hyalinosis, absence of leucocytic infiltration
Oguzturk, H., et al.,2016 [31]	In-vivo/ Rat	Cisplatin (7 mg/kg) & single injection & *ip*	↓Levels of SOD, GSH, & catalase, ↑Levels of TBARS, Esinophilic staining, ↑pyknosis of nuclei cells, hemorrhage & cell degeneration	50 mg/kg/day & for 14 consecutives days & by *po*	↑Levels of SOD, GSH, ↓Levels of TBARS, absence of esinophilic staining, pyknosis of nuclei cells, hemorrhage & cell degeneration
Al-Sefri, H. A., et al.,2020 [32]	In-vivo/ Rat	Doxorubicin (10 mg/Kg b.w) single dose & *ip*	↑Levels of cTn-I, cTn-T, FABP3, MYLK, CK-MB, LDH, AST, BNP, CRP, TNF-α, ox-LDL, & MDA, ↓SOD activity, ↑Cell degeneration, hemorrhage & vacuolization	50 mg/kg b.w & 30 days & *po*	↓Levels of cTn-I, cTn-T, FABP3, MYLK, CK-MB, AST, BNP, CRP, TNF-α, MDA, ↑SOD activity, Absence of cell degeneration, hemorrhage & vacuolization
Donia, T., et al.,2019 [33]	In-vivo/ Rat	Doxorubicin (4 mg /kg b.w) & three times per week for 2 weeks & *ip*	↑Levels of LDH, CK, TG, TC, LDL, MDA, NO, ↓Levels of HDL, GSH, ↑Activity of MPO, catalase, Caspase-3, ↓Activity of AE, & PON, Extensive loss of myofibril, ↑infiltration of inflammatory cells, vacuolization, ↑Apoptosis.	50 mg/kg b.w & three times per week for 3 weeks & *po*	↓Levels of LDH, CK, TG, TC, LDL, Heart MDA, NO ↑Levels of HDL, GSH ↓Activity of MPO, catalase, Caspase-3 ↑Activity of AE & PON, Well-preserved myocardium, ↓Loss of myofibrils
Jia, Y., et al.,2022 [34]	In-vivo/ mouse	Cisplatin (5 mg/kg) & two times per week for 1 week & *ip*	↑Levels of cTnI, CK, LDH, TNF-α, IL6, & MDA ↓Levels of SOD, catalase, & GSH, upregulation of Bax & Caspase-3, downregulation of Bcl-2, ↓Expression of p62 & Nrf2, ↑Expression of Keap1, ↑Infiltration of inflammatory cells, Apoptosis & cellular edema	100 mg/kg once a day & one week & *po*	↓Levels of cTnI, CK, LDH, TNF-α, IL6 & MDA ↑Levels of SOD, catalase, & GSH, downregulation of Bax & Caspase-3 upregulation of Bcl-2, ↑expression of p62 and Nrf2, ↓expression of Keap1, ↓Infiltration of inflammatory cells, Apoptosis & cellular edema
				300 mg/kg once a day & one week & *po*	↓Levels of cTnI, CK, LDH, TNF-α, IL6 & MDA, ↑Levels of SOD, catalase, & GSH downregulation of Bax & Caspase-3 upregulation of Bcl-2, ↑expression of p62 and Nrf2, ↓expression of Keap1, ↓Infiltration of inflammatory cells, Apoptosis & cellular edema
Kumar, S., et al.,2011 [35]	In-vivo/ Rat	Cyclophosphamide (200 mg/kg b.w) & single dose & *ip*	↑Levels of CK, ALT, AST, LDH, TNF-α, & MDA, ↓Levels of SOD, catalase, GPx, GST, & GSH.	100 mg/kg b.w & one week & *po*	↓Levels of CK, ALT, AST, LDH, TNF-α, & MDA, ↑Levels of SOD, catalase, GPx, GST, & GSH
Shrivastava, M., et al.,2013 [36]	In-vivo/ Rat	Cyclophosphamide (200 mg/kg b.w) & single dose & *ip*	↑Levels of CK, ALT, AST, LDH, & MDA.	100 mg/kg b.w & one week & *po*	↓Levels of CK, ALT, AST, LDH, & MDA
Farooq, J., et al.,2024 [37]	In-vivo/ Rat	5-Fluorouracil (150 mg/kg b.w) & single dose & *ip*	↑Levels of CK-MB, CK-NAC, LDH, MPO, MDA, NO, IL-6, IL-8, & TNF-α, ↓Levels of SOD, GSH, & catalase, severe cardiac cell damage, including necrosis and vacuolar changes in muscle fibers, fragmentation.	100 mg/kg & eight days & *po*	↓Levels of CK-MB, CK-NAC, LDH, MPO, MDA, NO, IL-6, IL-8, & TNF-α, ↑Levels of SOD, GSH, & catalase, mild hyperemia
Saad, S., et al.,2020 [38]	In-vivo/ Rat	Doxorubicin (15 mg/kg) single dose & *ip*	↑Levels of CK-MB, cTn-I, & MDA, ↓Levels of catalase, & SOD, ↑Activity caspase-3, ↓Heart weight, edema, loss of cellular boundaries, myocardial fibers disorganization, cytoplasmic ↑Vacuolization, & lymphocytic infiltration.	20 mg/kg & one week & *po*	↓Levels of CK-MB, cTn-I, & MDA ↑Levels of catalase, & SOD, ↓Activity caspase-3, ↓Pathological lesions without full reversal.

↑, Increase; ↓, Decease; &, And; *ip*, Intraperitoneal; *po*, Per os; CK, Creatine kinase; CK-MB, Creatine kinase isoenzyme-MB; LDH, Lactate dehydrogenase; AST, Aspartate aminotransferase; IFN-γ, Interferon γ; IL-1β, Interleukin 1β; TNF-α, Tumor necrosis factor α; GPx, Glutathione peroxidase; SOD, Superoxide dismutase; LPO: Lipid peroxidation; GSH: Reduced glutathione; TBARS, Thiobarbituric acid reactive substances; cTn-I, Troponins I; cTn-T, Troponins T; FABP3, Fatty acid-binding protein; MYLK, Myosin light-chain kinase I; BNP, B-type Natriuretic Peptide; CRP, C-reactive protein; ox-LDL, Oxidized low-density lipoproteins; MDA, Malondialdehyde; TG, Triglycerides; TC, Total cholesterol; LDL, low-density lipoproteins; NO, Nitric oxide; HDL, High density lipoprotein; GSH, Glutathione; MPO, Myeloperoxidase; AE, Arylesterase; PON, Paraoxonase; IL-6, Interleukin 6; Bcl-2, Anti-B-cell lymphoma-2; Bax, Anti-Bcl-2-associated X protein; ALT, Alanine transaminase; GST, Glutathione-s-transferase; NF-κB, Nuclear factor kappa B; CK-NAC, Creatine kinase-NAC; IL-8, Interleukin 8

**Table 2:** The characteristics Hesperetin of included studies

Author, year	Models	Chemotherapy medications (dosage) & Treatment protocol & Route of administration type	Outcomes of chemotherapy medications on cardiac tissue	Hesperetin dosage & protocol of usage & route of administration	Hesperetin co-administration outcomes
Trivedi, P. P., et al., 2011 [39]	In-vivo/ Rat	Doxorubicin (4 mg/kg bw) & once a week for 5 weeks & *ip*	↑Levels of MDA, ↑comet parameters (TL, TM, OTM, & % DNA), cell damage, TUNEL-positive cells, ↓Levels of GSH, ↑Expression of NF-κB, p38, & Caspase-3. Disorganization of the cellular structure and vacuolization in the heart.	100 mg/kg bw & 5 days a week for 5 weeks & *po*	↓Levels of MDA, ↑Levels of GSH, ↓comet parameters (TL, TM, OTM, & % DNA), cell damage, TUNEL-positive cells. ↓Expression of NF-kB, p38, & Caspase-3. Decreased damage in the cardiac cellular morphology
Pop Moldovan, A., et al., 2024 [40]	In-vivo/ mouse	Epirubicin (2 mg/kg) & 6 doses every other day, starting from the second day of the experiment & *ip*	↑Expression of Bax, Caspase-3, ↑TUNEL-positive nuclei, ↓Expression of Bcl-2 cytoplasmic vacuolization, myofibril loss, and disarray of fibers, inflammatory cell infiltration and a slight increase in interstitial collagen fibers, noticeable disorganization of the intermediate filaments, localized accumulation of lipids and collagen	100 mg/kg & 13 consecutive days, starting from the first day of the experiment & *po*	↓Expression of Bax & Caspase-3, ↑Expression of Bcl-2. ↓TUNEL-positive nuclei, the heart tissue of the group receiving both treatments largely resembled that of the control group.

↑, Increase; ↓, Decease; &, And; *ip*, Intraperitoneal; *po*, Per os; GSH: Glutathione; MDA, Malondialdehyde; Bcl-2, Anti-B-cell lymphoma-2; Bax, Anti-Bcl-2-associated X protein; NF-κB, Nuclear factor kappa B; TL, tail length; TM, tail moment; OTM, olive tail moment

### The role of hesperidin and Hesperetin against cardiotoxicity induced by chemotherapy medications

#### Doxorubicin

Doxorubicin is an anthracycline family employed as a powerful chemotherapeutic medication for the treatment of many malignancies. Nonetheless, its practical application is constrained by associated adverse effects, notably its cardiotoxicity [29]. The studies indicated that when doxorubicin was used, the levels of important antioxidants like glutathione peroxidase (GPx), glutathione (GSH), arylesterase (AE), catalase, superoxide dismutase (SOD), and paraoxonase (PON) were lower compared to the normal control group, indicating more oxidative stress. It was discovered that GPx, GSH, AE, catalase, SOD, and PON worked better when hesperidin was taken with doxorubicin compared to when doxorubicin was taken by itself [30–34]. Additionally, it was shown that giving doxorubicin led to a significant increase in the activity of nuclear factor kappa B (NF-κB), myeloperoxidase (MPO), p38, and caspase-3, as well as higher levels of oxidized low-density lipoproteins (ox-LDL), nitric oxide (NO), malondialdehyde (MDA), comet assay parameters (OTM, TM, TL, and % DNA), and TUNEL-positive cells compared to the normal control group. When hesperidin and doxorubicin were given together, the levels of MPO, ox-LDL, MDA, and NO, along with the activity of NF-κB, p38, and caspase-3, decreased significantly compared to the doxorubicin group. When hesperidin and doxorubicin were given together induced the levels of MPO, ox-LDL, MDA, and NO as well as activity of NF-κB, p38, and caspase-3 dropped significantly in comparison to the doxorubicin group [31, 33–35]. Contrary to the results of other studies, Donia, T., et al. identified elevated catalase activity in the doxorubicin group compared to the normal group which is reduced when co-administered with both hesperidin and doxorubicin [31].

It was found that giving doxorubicin greatly increased the levels of biomarkers for cardiac injury. Troponin I (cTn-I), troponin T (cTn-T), myosin light-chain kinase I (MYLK), lactate dehydrogenase (LDH), and aspartate aminotransferase (AST) were some of these that were studied. Compared to the normal control group, the doxorubicin group had higher levels of inflammatory markers like tumor necrosis factor α (TNF-α), interleukin 1β (IL-1β), interferon γ (IFN-γ), and C-reactive protein (CRP). When hesperidin and doxorubicin were given together, they greatly lowered levels of cTn-T, cTn-I, MYLK, LDH, AST, TNF-α, IL-1β, IFN-γ, and CRP compared to the doxorubicin group [30, 31, 33, 35].

High levels of brain natriuretic peptide (BNP) were seen after doxorubicin was given. BNP is a biomarker that shows the heart’s ventricular walls were stretching more than in the normal control group. When hesperidin and doxorubicin were given together, the higher BNP levels were restored compared to the doxorubicin group [35]. The heart tissue analysis showed that doxorubicin caused the heart to weigh more, increased inflammation, added collagen fibers, caused swelling, severe cell damage, disorganized heart cells, thickened heart muscle, created many small spaces in the cells, made blood vessel walls thicker, and led to too many red blood cells leaving the heart compared to the healthy control group.

When doxorubicin and hesperetin were injected together, they reduced damage to heart cells compared to the group that only got doxorubicin [30, 31, 33–35].

#### Paclitaxel

Paclitaxel is a diterpenoid that belongs to the Taxanes group. It is an anticancer drug that is used to treat cancers of the breast, lung, and ovary [36, 37]. Nonetheless, its practical application is hindered by its cardiotoxicity [38]. The study also found a link between paclitaxel induction and cardiac oxidative stress, including elevation of lipid peroxidation (LPO) levels, decreases in GSH level as well as activity of SOD and GPx. When paclitaxel and hesperidin were given together, they dropped the LPO level while raising SOD and GPx activity compared to the paclitaxel group. Moreover, the study’s findings show that giving paclitaxel increased heart function biomarkers, including CK-MB and LDH, compared to the healthy control group. Co-administration of paclitaxel with hesperidin significantly decreased the high levels of CK-MB and LDH. The hearts of mice that were given paclitaxel had a lot of hyalinosis and white blood cell infiltration in certain areas. Even so, when paclitaxel and hesperidin were given together, there was no white blood cell infiltration and a lot of hyalinosis became visible [39].

#### Cisplatin

Cisplatin is an antineoplastic agent based on platinum that is employed to treat multiple cancer types, including testicular, ovarian, head and neck, and lung malignancies [40]. Even though cisplatin is widely used and has strong anticancer properties, it has been linked to adverse effects, such as cardiotoxicity [41]. It was found that giving cisplatin to animals changed oxidative stress indicators in a big way, including making more thiobarbituric acid reactive substances (TBARS) and MDA. Moreover, there was a remarkable decrease in SOD, GSH, and catalase levels in comparison to the normal control group. The combined treatment of cisplatin and hesperidin brought the high levels of TBARs and MDA back to normal compared to the group that only received cisplatin. Furthermore, studies indicated that people who were given both cisplatin and hesperidin had higher levels of SOD, catalase, and GSH than people who were only given cisplatin [42, 43].

Cisplatin caused cardiotoxicity, which was linked to significantly higher levels of myocardial biomarkers like troponin I (cTnI), creatine kinase (CK), and LDH, as well as higher levels of inflammatory cytokines like TNF-α and interleukin-6 (IL-6), compared to the healthy control group. Even so, when hesperidin was given along with cisplatin, cardiac markers (cTnI, CK, and LDH) and inflammatory cytokines (TNF-α, IL6) were significantly lowered compared to the cisplatin group [43]. While comparing the cisplatin group to the control group, the study’s results showed that apoptotic factors like Bax and caspase-3 were turned up and anti-B-cell lymphoma-2 (Bcl-2) was turned down. Conversely, the co-administration of Hesperidin with Cisplatin resulted in a significant down-regulation of Bax and caspase-3 expression, whereas Bcl-2 expression was elevated in comparison to the Cisplatin group. On top of that, compared to the healthy control group, cisplatin-treated mice had much lower levels of signaling pathway proteins like p62 and Nrf2. On the other hand, they had much higher levels of Keap1. On the other hand, the study’s results indicated that cisplatin-treated animals had inflammatory cells, apoptotic cells, and edematous cardiac cells in their tissues, while the control group had a normal histological appearance. In animals administered Hesperidin in conjunction with cisplatin, the histological alterations were mitigated [42, 43].

#### Cyclophosphamide

Cyclophosphamide is a potent antineoplastic agent, classified as an oxazaphosphorine prodrug with alkylating capabilities. It is utilized in the management of a wide array of malignancies and autoimmune disorders [44, 45]. Cardiotoxicity is an adverse effect linked to cyclophosphamide [46]. This research shows that adding cyclophosphamide lowers the amounts of SOD, catalase, GPx, GST, GSH, and TNF-α compared to the healthy control group. In rats administered hesperidin alongside cyclophosphamide, a restoration of elevated levels of these biomarkers was seen.

When cyclophosphamide was given, levels of CK, ALT, AST, LDH, and MDA were all significantly higher than in the control group. When given with cyclophosphamide, hesperidin brought the elevated levels of these markers back to normal [47, 48].

#### Epirubicin

Epirubicin is an anthracycline agent that is a 4′ epimer of doxorubicin and works against different types of cancer [49]. Both epirubicin and doxorubicin are linked to cardiotoxicity; however, epirubicin demonstrates less cardiotoxicity [50]. The study’s findings demonstrated that epirubicin administration led to an increase in Bax expression, a significant rise in caspase-3 immunostaining, and a decrease in Bcl-2 expression, all of which are indicators of apoptosis, compared to the normal control group. Co-administration of epirubicin and hesperetin resulted in decreased levels of Bax and caspase-3, whereas Bcl-2 levels increased in comparison to the epirubicin-only group. The results of the current investigation indicated that epirubicin therapy significantly increased the quantity of TUNEL-positive nuclei compared to the control group. Animals treated with both epirubicin and hesperetin demonstrated a reduced count of positive nuclei relative to those provided with epirubicin alone.

According to the histological data, epirubicin treatment led to more cytoplasmic vacuolization, myofibril loss, fiber disorganization, infiltration of inflammatory cells, minor improvement of interstitial collagen fibers, significant disarray of intermediate filaments, and lower expression of desmin compared to the normal control group [51].

#### 5-Fluorouracil

5-Fluorouracil (5-FU) is a type of chemotherapy drug that belongs to the pyrimidine analogue group and is commonly used to treat different types of cancer. It inhibits the proliferation of cancer cells by inhibiting DNA and RNA synthesis [52].

In the Farooq, J., et al. study, it was found that 5-FU led to lower levels of oxidative stress markers SOD, GSH, and catalase compared to the normal control group. However, when hesperidin was given along with 5-FU, there was a significant increase in SOD, GSH, and catalase levels compared to animals that only received 5-FU. While co-administration of hesperidin and 5-FU showed a significant increase in SOD, GSH, and catalase levels compared to animals receiving only 5-FU. Also, 5-FU significantly increased the MPO, MDA, NO, IL-6, IL-8, and TNF-α levels compared to the control group, which is modulated by hesperidin. Furthermore, the levels of cardiac injury markers, including CK-MB, CK-NAC, and LDH, were notably increased by 5-FU treatment as compared to the control group. Concomitant administration of hesperidin and 5-FU led to a significant increase in MB, CK-NAC, and LDH levels compared to the 5-FU group [32].

In addition, histopathological investigations revealed that 5-FU administration is correlated with vacuolar alternations and necrosis in cardiac tissues of enrolled animals, leading to fragmentation and resembling severe cardiac cell injury compared to the control group. Animals treated with a combination of hesperidin and 5-FU were detected with mild hyperemia in histopathological studies [32].

## Discussion

The objective of the current investigation was to examine the processes underlying chemotherapy-induced cardiotoxicity and the effects of co-administering these medicines with hesperidin and hesperetin on cardiac function. Evidence indicates that chemotherapy medicines, while beneficial in cancer treatment, are linked to several negative effects across different organs. Cardiovascular adverse effects are particularly significant, as the heart is highly susceptible to these medications, and any cardiac damage may hinder the continuation of treatment [53].

**Fig. 2:**
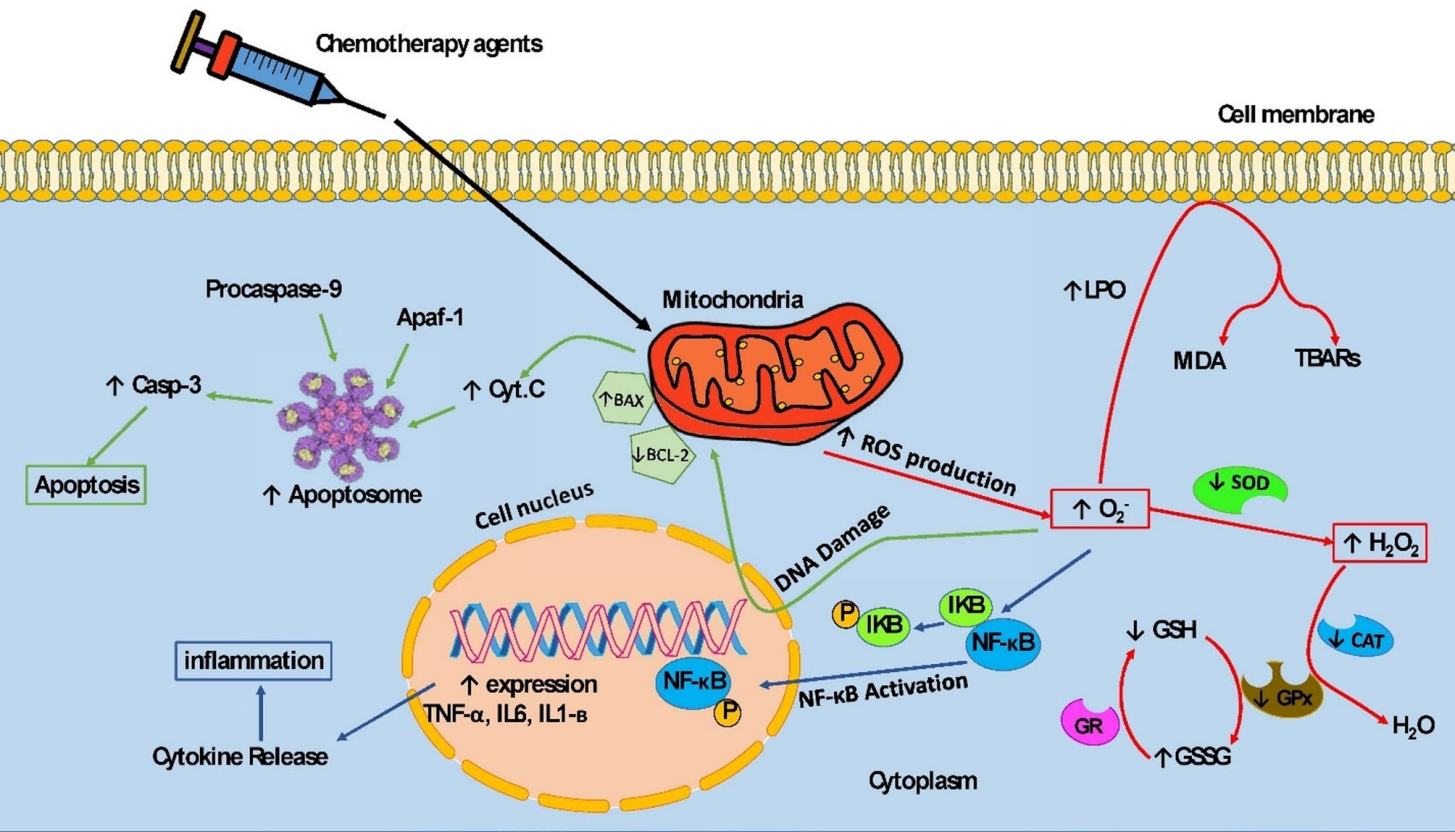
Mechanisms of chemotherapy-induced cardiotoxicity

Hesperidin and its aglycone, hesperetin, are naturally occurring chemicals that are present in several citrus fruits. Both hesperidin and hesperetin are thought to exert therapeutic effects on organs such as the heart and kidneys through several processes [18, 54]. This discussion reviews the impact of chemotherapeutic agents on cardiac cells and the functions of hesperidin and hesperetin. Figure 2 illustrates the main mechanisms of chemotherapy-induced cardiotoxicity and the role of hesperidin and hesperetin.

Chemotherapy agents trigger the formation of free radicals, leading to oxidative stress, inflammation, and the initiation of apoptosis. Hesperidin and hesperetin, through their protective properties, mitigate cardiotoxicity induced by chemotherapy agents.

↑Increased by chemotherapy; ↓Decreased by chemotherapy; phosphorylation (P); superoxide dismutase (SOD), catalase (CAT), glutathione peroxidase (GPx), glutathione reductase (GR), reduced glutathione (GSH), oxidised glutathione (GSSG), lipid peroxidation (LPO), malondialdehyde (MDA), thiobarbituric acid reactive substances (TBARs), apoptotic protease activating factor 1 (Apaf-1), cytochrome C (Cyt C), nuclear factor kappa-β (NF-κB), IkappaB (IKB), caspase 3 (Casp-3), Anti-B-cell lymphoma-2 (Bcl-2, ), Anti-Bcl-2-associated X protein (Bax), tumor necrosis factor-alpha (TNF-α), interleukin 6 (IL-6), and interleukin one beta (IL-1β).

### Anti-oxidant actions

While oxygen is necessary for aerobic respiration in humans, it also helps make compounds called activated oxygen or reactive oxygen species (ROS), which can damage cells’ functional and structural parts [55, 56]. Approximately 95% of oxygen is utilized for energy production and ultimately converted into water; however, the remaining 5% generates metabolites known as activated ROS, which exhibit high reactivity. ROS are created when molecular oxygen (O_2_) changes into superoxide anion radicals (O_2_⁻), hydroxyl radicals (HO⁻), hydrogen peroxide (H_2_O_2_), and other radicals [57, 58]. Consequently, ROS are standard physiological by-products of cellular metabolic processes, mostly generated by mitochondria [59]. Oxidative stress was caused by many physiological and pathological conditions, such as getting older, being active, low oxygen levels, radiation, and drug treatments (like chemotherapy medications) [60–63]. Researchers have found that normal levels of ROS are important for many cellular processes and act as signaling molecules [64, 65]. In addition, complex, multi-level antioxidant defense systems work to get rid of ROS or lessen the damage they do [66]. The oxidative stress pathway is made up of enzymes and molecules. These include the SOD, GPx, GR, and catalase enzymes, as well as GSH molecules, which are important for the antioxidant defense system [39, 42, 47, 67].

SOD enzyme serves as the primary defensive mechanism, transforming superoxide radicals into the less harmful hydrogen peroxide. GPx and catalase enzymes collaborate with SOD to decompose hydrogen peroxide in water [68–70]. During oxidative stress, GSH safeguards cells from harm inflicted by free radicals. GPx helps change GSH into glutathione disulfide (GSSG) in the ROS enzyme reaction. This procedure lowers the amounts of both GSH and ROS [71, 72]. In addition, LPO is a complex biochemical process in which free radicals, especially ROS, damage lipids inside cell membranes. This results in the oxidative degradation of lipids, yielding lipid peroxides and other detrimental consequences [73].

Chemotherapeutic medications can elevate ROS levels through three mechanisms: (1) Some chemotherapeutics, like doxorubicin, raise ROS levels by breaking down into unstable radicals. (2) They do this by increasing the activity of pro-oxidative enzymes like NADPH oxidase. (3) They do this by selectively decreasing the activity of cellular antioxidants like SOD [74].

Hesperidin and hesperetin are powerful antioxidants that work in two main ways: directly (by scavenging free radicals and giving hydrogen to free radicals) and indirectly (by stopping prooxidative enzymes, increasing antioxidant enzymes, protecting mitochondrial function, and improving glutathione redox status) [30, 31, 42, 47, 75, 76]. This study indicated that chemotherapy medications led to oxidative stress in the heart by decreasing the activity of SOD, GPx, catalase, and GST enzymes, increasing TBARS and MDA levels, and decreasing GSH levels. These effects were changed by hesperidin and hesperetin, which have antioxidant properties [30–35, 39, 42, 43, 47, 48].

### Anti-apoptotic actions

Apoptosis is a cautiously controlled process that gets rid of damaged and old cells. It helps keep things in balance and makes room for new, healthy cells [77, 78]. During apoptosis, cells’ structures change in several ways. In these processes, cells get smaller, chromatin condenses, membranes bleb, nuclei fragment (DNA laddering), apoptotic bodies form, and then neighboring cells phagocytize them [79, 80]. Research has indicated two primary molecular signaling mechanisms for apoptosis:

1. Intrinsic (mitochondrial) route; (2) extrinsic (death receptor) pathway [81]

Irradiation, oxidative stress, and treatment with cytotoxic drugs can all create stress inside cells that activate the intrinsic (mitochondrial) pathway. Bak and Bax are proteins belonging to the Bcl-2 family. When they attach to the mitochondrial membrane, it becomes leaky, letting cytochrome c move from the space between the membranes to the cytosol. After that, cytochrome c combines with procaspase-9 and apoptotic protease activating factor 1 (APAF-1) to form the apoptosome that activates caspase-9. Activation of caspase-9 initiates a cascade of caspase activation involving caspase-3, -6, and − 7. This results in the cleavage of cellular proteins, fragmentation of DNA, and subsequent cell death [56, 82, 83]. Extracellular death ligands interact with and turn on death receptors on the target cell’s surface to start the extrinsic (death receptor) pathway. These death ligands normally come from other cells, mainly immune cells or nearby cells, with varied goals, such as the eradication of contaminated or cancerous cells. Adaptor proteins, such as FADD (Fas-associated death domain), are utilized to assemble the death-inducing signaling complex (DISC) upon activation of certain death receptors. DISC subsequently activates procaspase-8, transforming it into caspase-8. The activation of caspase-8 initiates a cascade of caspase activations, including caspases 3, 6, and 7. This induces DNA fragmentation, cleavage of cellular proteins, and ultimately results in apoptosis [84–86].

Chemotherapeutic agents can induce apoptosis in cancer cells, which display an unusually high rate of division. Chemotherapy efficiently promotes death in cancer cells; nevertheless, it also affects healthy cells, leading to various side effects and organ damage [87–90]. Chemotherapeutic agents such as doxorubicin induce apoptosis in healthy cardiac cells. This phenomenon occurs in both acute and chronic myocyte loss, ultimately leading to cardiomyopathy [91, 92]. Thus, they can induce apoptosis by engaging both intrinsic and extrinsic pathways [31, 33, 34, 42, 43, 51]. Hesperetin and hesperidin also show significant anti-apoptotic effects, as the current study shows. When you give hesperetin and hesperidin, DNA damage goes down and caspase-3 activity goes up [31, 33, 34, 43, 51]. The activity of the NF-κB transcription factor is also decreased when hesperidin and hesperetin are given. This chemical enhances the activity of the Bcl-2 family while diminishing the activity of Bax [34, 43, 51].

### Anti-inflammatory actions

Inflammation is a multifaceted reaction initiated by the immune system to potentially damaging stimuli, including infections, injury, and metabolic stress, to maintain tissue homeostasis [93, 94]. The primary characteristics of inflammation encompass erythema, edema, increased temperature, dolor, and impairment of tissue function [95]. From a microscopic viewpoint, immune cells trigger an inflammatory response by identifying primary damaging stimuli and, subsequently, activating various intracellular signaling pathways inside the immune cells. The outcome of these pathways’ activation is the stimulation of transcription factors, including NF-κB and AP-1. Active transcription factors facilitate the expression of inflammatory genes. This produces a diverse array of inflammatory mediators, including cytokines (such as IL-1β, IL-6, IL-10, IFN-γ, and TNF-α), chemokines, prostaglandins, and leukotrienes. These mediators bring neutrophils, monocytes, and lymphocytes to the site of the first stimulus, where they kill the first damaging stimuli by phagocytosis or cytotoxicity [96, 97]. Nevertheless, these activities may adversely affect healthy cells at the inflammatory site, potentially resulting in significant side effects and organ damage [98]. Numerous studies have revealed the chemotherapeutic medication’s inflammatory effects on the heart [99]. Researchers have found that chemotherapeutic medications induced elevation in levels of IFN-γ, TNF-α, IL-6, IL-8, CRP, IL-1β, and Nrf2. This elevation was significantly modified by the administration of hesperidin and hesperetin in the heart [30, 32, 35, 43, 47]. In addition, researchers have found that giving hesperidin and hesperetin lowers the activity of pro-inflammatory enzymes like cyclooxygenase-2 (COX-2), which makes prostaglandins. It also obstructs the activation of the NF-κB pathway, leading to the attenuation of inflammation [99, 100].

## The perspective of future research

Chemotherapy is a prevalent cancer treatment globally, utilizing diverse effective cytotoxic medications with distinct modes of action to inhibit cells exhibiting aberrant proliferation and division rates. While chemotherapy significantly extends the lives of cancer patients, it still poses significant challenges to their quality of life. Chemotherapy chemicals can damage healthy cells due to their cytotoxic properties [8]. Moreover, the manifestation of these adverse effects can hinder clinicians from persisting with treatment and diminish the efficacy of chemotherapy. Cardiotoxicity is a significant adverse consequence of chemotherapy, presenting in numerous manifestations, such as arrhythmias, alterations in blood pressure, myocarditis, heart failure, and sudden myocardial infarction [101]. A randomized controlled trial (RCT) systematic review and meta-analysis research on 525 participants have examined the efficacy of hesperidin for various illnesses. Hesperidin supplements have been shown to lower serum levels of triglycerides, total cholesterol, and low-density lipoprotein. Furthermore, hesperidin lowers TNF-α and blood pressure, which may affect a patient’s cardiovascular risk factors [102]. Moreover, in another RCT study, hesperidin significantly improved patients with metabolic syndrome and diabetic neuropathy [103]. Also, hesperidin reduced systolic blood pressure and tended to lower diastolic blood pressure in type 2 diabetes, according to another RCT systematic review and meta-analysis study that included 656 patients [104]. The present study, based on animal and in vitro research, indicates the positive effects of hesperidin and hesperetin on cardiotoxicity induced by chemotherapy. The therapeutic potential of hesperidin and hesperetin in mitigating cardiotoxicity induced by chemotherapy remains inadequately understood and warrants further investigation through rigorously designed clinical trials to elucidate their efficacy and mechanisms of action.

## Conclusion

This study’s findings underscore that cardiotoxicity is among the most perilous adverse effects induced by chemotherapy agents. Chemotherapy adversely affects the heart by generating damaging ROS, initiating apoptosis via several pathways, and inducing inflammation. Hesperidin and hesperetin have been shown to lower the risk of cardiotoxicity when used with chemotherapy medications. This beneficial effect is achieved by alleviating oxidative stress, preventing apoptosis, and modifying the processes that govern inflammation. Based on research that hasn’t been done yet, this study suggests that using hesperidin and hesperetin together might improve chemotherapy management. The results highlight the translational potential of these flavonoids, emphasizing the need for thorough preclinical optimization and clinical trials to confirm their efficacy and safety. This review establishes a basis for incorporating natural chemicals into cardioprotective approaches in oncology. Further well-designed clinical trials are necessary to confirm the cardioprotective effects of hesperidin and hesperetin.

## Data Availability

No datasets were generated or analysed during the current study.
